# Predictors of Internet Health Information–Seeking Behaviors Among Young Adults Living With HIV Across the United States: Longitudinal Observational Study

**DOI:** 10.2196/18309

**Published:** 2020-11-02

**Authors:** Warren Scott Comulada, Mary Step, Jesse B Fletcher, Amanda E Tanner, Nadia L Dowshen, Sean Arayasirikul, Kristin Keglovitz Baker, James Zuniga, Dallas Swendeman, Melissa Medich, Uyen H Kao, Adam Northrup, Omar Nieto, Ronald A Brooks, Special Projects Of National Significance Social Media Initiative Study Group

**Affiliations:** 1 Department of Psychiatry and Biobehavioral Sciences University of California, Los Angeles Los Angeles, CA United States; 2 Department of Health Policy and Management University of California, Los Angeles Los Angeles, CA United States; 3 College of Public Health Kent State University Kent, OH United States; 4 Friends Research Institute, Inc Los Angeles, CA United States; 5 Department of Public Health University of North Carolina Greensboro Greensboro, NC United States; 6 Division of Adolescent Medicine Children's Hospital of Philadelphia Philadelphia, PA United States; 7 Department of Pediatrics University of California, San Francisco San Francisco, CA United States; 8 Howard Brown Health Center Chicago, IL United States; 9 Center for the Study of Healthcare Innovation, Implementation and Policy, Health Services Research & Development Veterans Affairs Greater Los Angeles Health Care System US Department of Veteran Affairs Los Angeles, CA United States; 10 Department of Family Medicine University of California, Los Angeles Los Angeles, CA United States; 11 See Acknowledgments

**Keywords:** youth, young adults, HIV/AIDS, digital technology, health information seeking, general health, sexual and reproductive health, HIV stigma

## Abstract

**Background:**

Consistent with young adults’ penchant for digital communication, young adults living with HIV use digital communication media to seek out health information. Understanding the types of health information sought online and the characteristics of these information-seeking young adults is vital when designing digital health interventions for them.

**Objective:**

This study aims to describe characteristics of young adults living with HIV who seek health information through the internet. Results will be relevant to digital health interventions and patient education.

**Methods:**

Young adults with HIV (aged 18-34 years) self-reported internet use during an evaluation of digital HIV care interventions across 10 demonstration projects in the United States (N=716). Lasso (least absolute shrinkage and selection operator) models were used to select characteristics that predicted whether participants reported seeking general health and sexual and reproductive health (SRH) information on the internet during the past 6 months.

**Results:**

Almost a third (211/716, 29.5%) and a fifth (155/716, 21.6%) of participants reported searching for general health and SRH information, respectively; 26.7% (36/135) of transgender young adults with HIV searched for gender-affirming care topics. Areas under the curve (>0.70) indicated success in building models to predict internet health information seeking. Consistent with prior studies, higher education and income predicted health information seeking. Higher self-reported antiretroviral therapy adherence, substance use, and not reporting transgender gender identity also predicted health information seeking. Reporting a sexual orientation other than gay, lesbian, bisexual, or straight predicted SRH information seeking.

**Conclusions:**

Young adults living with HIV commonly seek both general health and SRH information online, particularly those exploring their sexual identity. Providers should discuss the most commonly sought SRH topics and the use of digital technology and be open to discussing information found online to better assist young adults with HIV in finding accurate information. Characteristics associated with health information–seeking behavior may also be used to develop and tailor digital health interventions for these young adults.

## Introduction

Interventions for young adults living with HIV (YALH) increasingly capitalize on the popularity and integration of digital communication media into daily routines. Social media use has saturated the information landscape in the United States, with near-ubiquitous social media platform use among those younger than 30 years [1]. Consistent with their heavy use of digital forms of communication, youth and young adults use digital communication media to seek out personalized and pertinent health information [2]. Though an income-based digital divide persists [3], reliance on electronic health information is common among youth from marginalized populations, including those living with HIV [4] and unstably housed [5]. In fact, almost half (47%) of runaway and homeless youth sought information about HIV or other STIs, and 40% sought information about sex or sexuality from online sources [5].

While the penchant for digital communication among youth and young adults can be harnessed to develop digital health interventions (eg, interventions that provide health information through online sources, social media, and text messages), these strategies are challenged by the growing levels of health misinformation available from digital sources [6]. Younger people often need guidance engaging with accurate health information designed for them. Understanding the types of health information sought online and the characteristics of these information-seeking young adults, especially vulnerable or stigmatized populations, is vital when designing digital health interventions to reach these communities.

Prior studies on internet health information seeking have focused on the general population and patient populations not living with HIV, mostly adults. These studies demonstrated that certain characteristics, including greater socioeconomic stability [7,8], more internet experience [9], female gender [8], less perceived social support [10], better health care provider relationships, and greater health engagement are associated with greater online health information seeking [11]. Behavioral characteristics, including poorer mental or physical health and alcohol and tobacco use also correlate with internet health information seeking [7,8,12]. Importantly, Mitchell et al [13] found that sexual minority youth are more likely to use the internet to seek sexual health information than their heterosexual counterparts.

In one notable addition to the literature, Calvert et al [14] evaluated adults living with HIV and found that greater socioeconomic stability was associated with greater engagement with online health information seeking. Studies focused specifically on how YALH seek health information are needed because the stigma associated with HIV is a known barrier to care [15]. Stigma and the intersectionality of multiple marginalized identities (racial/ethnic minority, gender identity, sexual orientation) of many YALH may represent opportunities for safe exploration and information seeking through digital spaces [16]. Furthermore, a robust examination of predictive models of internet health-seeking behaviors will provide valuable information for tailoring digital interventions to YALH.

To address this goal, analyses for this paper applied machine learning (ML) methods to data from a digital health intervention initiative for YALH to identify salient characteristics that predict internet health-seeking behaviors. The comprehensive model of information seeking [17] and correlates of online health seeking from prior studies provided a framework for selecting candidate predictors. An individual’s sociodemographic characteristics were conceptualized as preceding, and even influencing, where people seek information [18]. Information seeking refers to intentional efforts made by individuals to satisfy their information needs or goals [18], such as HIV-related health care needs. As a secondary aim, the study evaluated individual predictors selected by ML methods and compared these findings with extant literature.

## Methods

### Participants

Data used for this analysis were collected as part of an initiative funded by the Health Resources and Services Administration to evaluate digital health interventions targeting young people living with HIV (aged 13 to 34 years) across 10 demonstration sites in the United States. Digital interventions were developed by each site and varied in content and delivery format, which included automated text messaging, mobile apps, and social media. Common intervention elements included health promotion messages, enhanced communication with intervention staff using digital communication tools, and HIV medication and medical care appointment reminders. All interventions targeted HIV care continuum outcomes and were evaluated over 18 months.

Young people living with HIV were recruited in the following cities from October 2016 through May 2018: Chicago, Illinois; Cleveland, Ohio; Corpus Christi, Texas; Hershey, Pennsylvania; Los Angeles, California; New York, New York; Philadelphia, Pennsylvania; San Francisco, California; St Louis, Missouri; and Winston-Salem, North Carolina. Recruitment took place through community and university clinics, health departments, a hospital system, and a community research site. Eligibility for study enrollment required young people to have a confirmed HIV diagnosis, be between the ages of 13 and 34 years, be capable of filling out audio computer-assisted self-interview (ACASI) assessments administered in English or Spanish, and meet at least one of the following criteria based on the US Department of Health and Human Services (HHS) common core indicators for monitoring HHS-funded HIV care services: (1) newly diagnosed with HIV within the last year upon enrollment, (2) not newly diagnosed and not currently engaged in HIV care, (3) never linked to HIV medical care, regardless of the duration of HIV infection, and (4) not virally suppressed, defined as having a viral load of 200 copies/mL or greater. Demonstration sites had additional eligibility criteria, such as being a patient at the site’s clinic or owning a smartphone, if required by their digital health intervention. Participants from all genders, races and ethnicities, and sexual orientations were included in the initiative. Details on the initiative and the intervention typology across sites are described in Medich et al [19].

**Figure 1:**
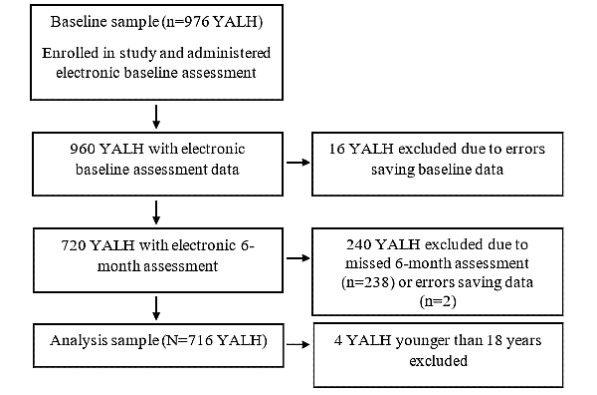
Sample selection process for analyses using baseline data to predict internet health information–seeking behaviors assessed 6 months after baseline. YALH: young adults living with HIV.

Figure 1 shows the process used to select participants for analysis. Analyses in this paper incorporated predictors measured at baseline and outcomes measured at 6 months post enrollment (N=720 participants). Participants with missing baseline or 6-month assessments were excluded. Missing data occurred from errors saving electronic assessment files or missing assessments (eg, due to attrition after the baseline assessment). There were only 4 participants younger than 18 years old, making it difficult to model health-seeking behaviors in this group. Moreover, the younger participants represented a different patient population in terms of clinical practice. Therefore, they were excluded, and the final analytical sample contained 716 YALH.

### Procedure

All data collection procedures for the cross-site evaluation were approved by the institutional review board at the University of California, Los Angeles (UCLA; No. 15-001625), the institution that was responsible for collecting and evaluating data across the sites. At enrollment, each site screened, consented, and administered a baseline ACASI assessment to participants using Questionnaire Design Studio software (Nova Research Company). Sites also collected participants’ medical chart data, either by hand abstraction or from administrative records associated with the receipt of Ryan White HIV/AIDS Program funds. ACASI assessments were administered and medical chart data were obtained by sites every 6 months over the 18-month follow-up period. Sites submitted deidentified ACASI and medical chart data to the UCLA evaluation center through a web-based secure portal.

### Measures

Measures treated as predictors were assessed at baseline. After baseline assessment began, measures that better captured evolving trends in technology usage among lesbian, gay, bisexual, transgender, and queer or questioning youth than baseline measures were developed and added to the 6-month follow-up assessment. These measures are treated as outcomes in the analyses.

### Predictors Measured at Baseline

#### Sociodemographic Characteristics

Age was calculated from the self-reported month and year of birth. Participants were asked to specify the race with which they identified and indicate whether they were Hispanic or Latinx. They designated their current gender identity with categories for male, female, transgender man, transgender woman, genderqueer or nonconforming, or other gender identity. Participants were also asked to categorize their sexual orientation as straight, lesbian or gay, bisexual, queer, other, or don’t know/not sure; responses indicating “other” varied, included pansexual, nonsexual, and refusals to answer. Participants specified whether they were currently in school and the highest level of education they had completed. They were also asked to report monthly income “from all sources combined” and their current employment status (eg, full-time, part-time, student, or disabled). Housing stability was assessed by asking participants to indicate which type of place they stayed in the most in the past week (eg, a house or homeless shelter).

#### Region

Most transgender women were recruited in Los Angeles, since the Los Angeles site intervention targeted transgender women. Collinearity that would have resulted by including site and gender identity as predictors was addressed by replacing site with a predictor based on Census Bureau regions for the United States. Categories were created for the West (Los Angeles and San Francisco, California), Midwest (Chicago, Illinois; Cleveland, Ohio; and St Louis, Missouri), South (Corpus Christi, Texas, and Winston-Salem, North Carolina), and Northeast regions (Hershey and Philadelphia, Pennsylvania, and New York, New York).

#### Health Insurance

Participants were asked what type of health insurance they had. Insurance status was dichotomized as being insured versus not being insured or not knowing one’s insurance status.

#### Time Since HIV Diagnosis

Time since HIV diagnosis was calculated as the number of years between the self-reported HIV diagnosis date and the baseline assessment date.

#### Antiretroviral Therapy Adherence

Antiretroviral therapy (ART) adherence was assessed using the self-rating scale item [20,21]. Categories were collapsed to indicate low (“very poor” or “poor”), neutral (“fair”), or high adherence (“good”, “very good”, or “excellent”).

#### Viral Load

Viral load data were obtained via abstraction from patient medical records. Viral load was categorized as suppressed (at less than 200 copies/mL), unsuppressed, or missing. A missing data category was included because sites were unable to obtain medical chart data on all participants.

#### Doctor’s Office Visitation

Similar to viral load, HIV-related ambulatory care visit attendance was obtained from medical record data. Attendance was categorized as having had an HIV medical visit in the past 6 months, not having had an HIV visit in the past 6 months, or missing.

#### Health Engagement

Health engagement was also assessed through the youth engagement with health services (YEHS) survey [22]. Responses were summed across 2 YEHS subscales for health access literacy (5 items; Cronbach α=.81) and health self-efficacy (5 items; Cronbach α=.89).

#### Provider Empathy

The consultation and relational empathy measure was used to assess participants’ perceptions of health care provider empathy (10 items; Cronbach α=.98) [23].

#### Substance Use

Participants were asked to indicate any nonprescribed substances they used but did not inject in the past 6 months from a checklist (ie, recent use). Both proper names and street names of substances were presented in the checklist, such as methamphetamine and “Tina.” Indicator variables were created to denote use (1) or nonuse (0) for alcohol, tobacco, marijuana, and other substances, such as synthetic marijuana, methamphetamine, cocaine, heroin, and painkillers. Other substances were not modeled separately due to self-reported rates of use that were less than 10%, except for methamphetamines (118/720, 16.3%), inhalants (113/720, 15.7%), and powder cocaine (88/720, 12.2%). Participants were asked about lifetime and recent injection drug use, excluding prescribed medications.

#### Perceived Confidence in Receiving Social Support From Family and Friends

Perceived social support availability from family and friends was assessed through 3 social support items from the coping self-efficacy scale (3 items; Cronbach α=.83) [24], in which participants were asked about confidence in receiving support from family and friends on a scale from 0 (not confident at all) to 10 (very confident).

#### HIV Status Disclosure

HIV status disclosure was dichotomized as disclosure to one or more individuals or to none based on the participant response to having ever told anyone that they have HIV. If they had disclosed their status, they were asked to indicate types of individuals to whom they disclosed their HIV status (eg, partners and family members).

#### HIV-Related Stigma

HIV-related stigma was assessed through the revised HIV stigma scale (10 items; Cronbach α=.89) [25]. Using a scale from 1 (strongly disagree) to 4 (strongly agree), respondents were asked to rate their agreement with statements about experiencing HIV stigma.

#### General Physical and Mental Health

General physical and mental health quality of life was assessed with 4 questions from the 12-item Short-Form Health Survey [26]. Participants were asked if they “felt calm and peaceful,” had “a lot of energy,” or “felt downhearted and blue” over the past 4 weeks. The 3 items were summed to create a mental health measure (Cronbach α=.66). Participants were also asked how often their physical health or emotional problems interfered with social activities.

#### The Media and Technology Usage and Attitudes Scale

The Media and Technology Usage and Attitudes Scale (MTUAS) subscales [27] were administered to assess the frequency of digital communication use, including emailing (4 items; Cronbach α=.88), texting (4 items; Cronbach α=.67), smartphone use (9 items; Cronbach α=.90), internet searching (4 items; Cronbach α=.91), and general social media use (9 items; Cronbach α=.92). Subscales on positive attitudes toward technology (6 items; Cronbach α=.88), anxiety about being without technology or dependence on technology (3 items; Cronbach α=.88), and negative attitudes toward technology (3 items; Cronbach α=.81) were also administered.

#### Outcomes Measured at 6 Months Post Enrollment

Participants were asked what types of digital media and communication tools they used and what types of information were sought and discussed. For this analysis, the focus centered on questions that queried the types of information that were sought through the internet. Sexual health information (eg, practicing safer sex and HIV information) discussed or sought through text messaging, email, private messaging, and social networking applications is also presented to describe the sample. Two binary outcome measures were created for (1) having looked up general health (GH) information on the internet in the past 6 months and (2) having looked up sexual and reproductive health (SRH) information on the internet in the past 6 months. Transgender health information seeking (eg, gender-affirming hormone information) was also assessed, but rates were too low to analyze using ML models.

### Statistical Analysis

All analyses were conducted using R software (version 3.5.3; R Project for Statistical Computing) [28]. Data were randomly split into training (537/720, 74.6%) and testing data sets (179/720, 24.9%). A ML approach was chosen to meet the aims of the paper to build a predictive model and evaluate individual predictors selected by the model. In this vein, we used lasso (least absolute shrinkage and selection operator) regression as the ML approach because it fits a model to all candidate predictors and shrinks regression coefficients to zero for predictors that do not adequately contribute to error minimization. In other words, lasso regression provides a distinguishable subset of predictors, in contrast to ridge regression, which does not constrain regression coefficients to be zero, or to other ML approaches that provide less interpretable parameter estimates, such as random forest algorithms. The glmnet R package [29] was used to fit lasso logistic regression models to the training data set using 10-fold validation to select predictors for seeking general health information and SRH information via the internet.

Accuracy of the lasso models was gauged by using parameter estimates to predict internet health information seeking in the test data and comparing predictions to observed outcome values. Receiver operating curves (ROCs) were plotted to evaluate the sensitivity and specificity of predictions over a range of probability thresholds. Areas under the ROC curve (AUCs) are presented to gauge the accuracy of predictions. An AUC of 0.50 indicates a model that performs no better than chance.

Traditional logistic regressions were fit to the training data using predictors selected by lasso models to aid interpretation of associations between predictors and internet health seeking. Odds ratios (ORs) are reported. Statistical significance levels are not presented due to difficulties interpreting regression coefficient *P* values for subsets of predictors selected using ML algorithms.

## Results

### Sample Characteristics

Tables 1 and 2 show variables that were evaluated as candidate predictors of seeking health information on the internet across the 10 demonstration sites (N=716). Two-thirds of the participants were aged 25 to 34 years (483/716, 67.5%). Half of the participants reported a non-Latinx African American racial/ethnic identity (362/716, 50.6%); 27.9% (200/716) reported Latinx ethnicity. Most participants reported male gender (506/716, 70.7%). Nearly one-fifth (130/716, 18.2%) of participants identified as transgender women, and 5 of the 716 participants identified as transgender men (.01%). Approximately half of the participants identified as gay or lesbian (393/716, 54.9%), and half reported having no more than a high school education and access to stable housing (368/716, 51.4% and 363/716, 50.7%, respectively). The median monthly income was US $800 (IQR US $200 to $1500).

**Table 1:** Sociodemographic and HIV-related health care measures entered into lasso models as internet health-seeking predictors.

Characteristic		Values
**Site, n (%)**		
	Chicago	84 (11.7)
	Cleveland	82 (11.5)
	Corpus Christi	86 (12.0)
	Hershey	36 (5.0)
	Los Angeles	110 (15.4)
	New York	30 (4.2)
	Philadelphia	28 (3.9)
	St Louis	84 (11.7)
	San Francisco	88 (12.3)
	Winston-Salem	88 (12.3)
**Age group (years), n (%)**		
	18-24	233 (32.5)
	25-34	483 (67.5)
**Race/ethnicity, n (%)**		
	Latinx	200 (27.9)
	Non-Latinx African American	362 (50.6)
	Non-Latinx White	121 (16.9)
	Other racial/ethnic identity	33 (4.6)
**Gender identity, n (%)**		
	Male (ie, cisgender man)	506 (70.7)
	Female (ie, cisgender woman)	57 (8.0)
	Transgender-identified	135 (18.9)
	Other gender identity	18 (2.5)
**Sexual orientation, n (%)**		
	Straight	168 (23.5)
	Gay or lesbian	393 (54.9)
	Bisexual	99 (13.8)
	Other sexual orientation	56 (7.8)
**Education, n (%)**		
	High school/GED^a^ or less	368 (51.4)
	Some college education	220 (30.7)
	College degree or trade certification	128 (17.9)
**Current residence, n (%)**		
	Stable housing	363 (50.7)
	Unstable housing^b^	353 (49.3)
Monthly income, median (IQR)^c^		800 (200-1500)
**Health insurance status, n (%)**		
	Insured	489 (68.3)
	Not insured or don’t know	227 (31.7)
**HIV diagnosis, n (%)**		
	Within past 12 months	229 (32.0)
	>12 months	483 (67.5)
	Don’t know	4 (0.6)
**ART^d^ adherence, n (%)**		
	High	362 (50.6)
	Neutral	67 (9.4)
	Low	57 (40.1)
	Not on ART	230 (32.1)
Viral suppression^e^, n (%)		270 (42.3)
Recent doctor’s office visit^f^, n (%)		557 (86.9)
**Youth health engagement, mean (SD)**		
	Health access literacy (1-16^g^)	12.0 (3.5)
	Health self-efficacy (2-20^g^)	16.8 (4.0)
Provider empathy (10-50^g^), mean (SD)		42.6 (9.8)

^a^GED: general education development.

^b^Unstably housed group includes 6 participants who reported being hospitalized or in prison.

^c^N=655 due to “don’t know/not sure” responses.

^d^ART: antiretroviral therapy.

^e^Viral suppression information obtained from medical chart data (N=638).

^f^HIV doctor’s visit information obtained from medical chart data (N=641).

^g^Minimum and maximum values for scales are shown in parentheses.

Two-thirds of the participants reported being diagnosed with HIV for over a year (483/716, 67.5%) and having health insurance (489/716, 68.3%). Based on medical chart data, most participants had visited a doctor’s office for HIV in the past 6 months (557/641, 86.9%), but less than half of the participants were virally suppressed (270/641, 42.1%). Most participants disclosed their HIV status to someone (654/716, 91.3%), including partners (444/716, 62.0%) and friends or family (551/716, 77.0%). Two-thirds of the participants drank alcohol (490/716, 68.4%) and a little over half used marijuana (422/716, 58.9%) and tobacco (373/716, 52.1%) within the past 6 months. Less than half of the participants reported using other noninjected substances (320/716, 44.7%). Most participants used alcohol or at least one noninjectable substance within the past 6 months (598/716, 83.5%). A total of 16.2% (115/711) of the participants reported injection drug use.

Almost a third of the participants reported searching for GH topics on the internet over the past 6 months (211/716, 29.5%), followed by SRH information (155/716, 21.6%). In regard to discussing or seeking SRH through other digital communication tools, rates were 20.0% (143/716) for text messaging, 10.2% (73/716) for email, 8.7% (62/716) for social networking apps (eg, Facebook or Instagram), and 7.8% (56/716) for private messaging. Nearly a quarter of transgender participants reported searching for transgender-specific topics (36/135, 26.7%), including hormone therapy (30/135, 22.2%) and surgeries (24/135, 17.8%).

**Table 2:** Substance use, HIV-related disclosure and stigma, physical and mental health, and Media and Technology Usage and Attitudes Scale measures entered into lasso models as internet health-seeking predictors.

Characteristic		Values
**Recent substance use, n (%)**		
	Alcohol	490 (68.4)
	Tobacco	373 (52.1)
	Marijuana	422 (58.9)
	Other noninjected drugs	320 (44.7)
Lifetime injection drug use^a^, n (%)		115 (16.2)
HIV status disclosure, n (%)		654 (91.3)
HIV-related stigma (10-40^b^), mean (SD)		24.5 (7.5)
Perceived social support (0-30^b^), mean (SD)		21.0 (7.7)
**Physical and mental health, mean (SD)**		
	Mental health (3-18^b^)	9.4 (3.6)
	Mental and physical health (1-6^b^)	4.0 (1.7)
**MTUAS^c^, mean (SD)**		
	Email usage (0-36^b^)	19.8 (11.0)
	Text messaging (0-27^b^)	21.4 (6.0)
	Smartphone usage (0-81^b^)	62.1 (18.3)
	Internet searching (0-81^b^)	27.2 (9.8)
	General social media usage (0-81^b^)	49.1 (25.0)
	Positive technology attitudes (6-30^b^)	24.0 (5.1)
	Anxiety without or dependent on technology (3-15^b^)	10.9 (3.4)
	Negative technology attitudes (3-15^b^)	9.4 (3.1)

^a^N=711 for injected drugs due to refusal responses.

^b^Minimum and maximum values for scales shown in parentheses.

^c^MTUAS: Media and Technology Usage and Attitudes Scale.

### Predictors of Internet Health Information Seeking

**Figure 2:**
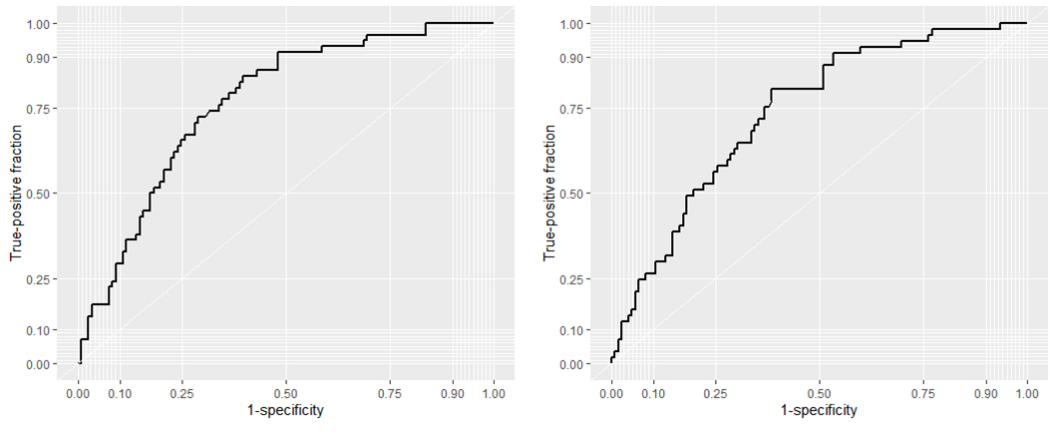
Receiver operating curves showing performance of lasso-selected predictors to predict general health information (left) and sexual and reproductive health information seeking (right) for different probability thresholds. Lasso: least absolute shrinkage and selection operator.

Figure 2 shows the ROCs for lasso-based predictions of GH and SRH information seeking in the test data. Curves above the 45° line indicate a degree of predictability beyond chance. AUCs for lasso models fit to GH information–seeking and SRH information–seeking outcomes are 0.76 and 0.73, respectively. To aid interpretation, we describe the accuracy of the model for a probability threshold of 0.50, where we classified participants as having searched the internet for health information if the predicted probability was greater than 0.50. A total of 32.4% (58/179) of the participants in the test data sought GH information on the internet. We correctly classified 16 as having sought GH information and correctly classified 112 of the 121 participants who did not seek GH information. Based on a 0.50 threshold, the accuracy of the GH model was (16 + 112) / 179 = 71.5%. Using the same formula, the accuracy of the SRH model at the 0.50 threshold was 70%.

Table 3 and 4 show ORs from logistic models fit to internet GH information–seeking and SRH information–seeking outcomes. Covariates are predictors selected from each lasso model. Mostly consistent with our hypotheses, having a high school degree or less was associated with lower odds of seeking GH and SRH information on the internet relative to having a higher degree (OR 0.49 and 0.68, respectively). Reporting high monthly income was associated with higher odds of seeking SRH information on the internet relative to no, low, or unreported monthly income. In a contradictory fashion, reporting low monthly income was associated with lower odds of seeking SRH information on the internet relative to no or unreported monthly income (OR 0.59). Participants reporting recent use of alcohol, tobacco, and marijuana had higher odds of seeking GH and SRH information (OR 1.29-1.70).

**Table 3:** Odds ratios from logistic regressions of general health information seeking through the internet over the past 6 months. Predictors were selected by lasso regression models.

Predictor		OR^a^
**Gender**		
	Male	1.19
	Transgender-identified	0.41
	Female (cisgender woman) or other^b^	N/A^c^
**Education**		
	High school or less	0.49
	Some college, college degree, or trade certificate^b^	N/A
**Monthly income**		
	Low (under median)	0.66
	None, don’t know, or high (median or above)	N/A
**ART^d^ adherence**		
	High	1.62
	Not using, low, or neutral	N/A
**Substance use^e^**		
	Alcohol	1.47
	Tobacco	1.36
	Marijuana	1.45

^a^OR: odds ratio.

^b^Reference category.

^c^N/A: not applicable.

^d^ART: antiretroviral therapy.

^e^Reference category was none for each substance.

**Table 4:** Odds ratios from logistic regressions of sexual and reproductive health information seeking through the internet over the past 6 months. Predictors were selected by lasso regression models.

Predictor		OR^a^
**Gender**		
	Male	1.26
	Transgender-identified	0.50
	Female (cisgender woman) or other^b^	N/A^c^
**Sexual orientation**		
	Other	2.48
	Straight, gay, lesbian, or bisexual^b^	N/A
**Race/ethnicity**		
	White	1.68
	Latinx, non-Latinx, Black, or Other^b^	N/A
**Education**		
	High school or less	0.68
	College degree or trade certificate	1.45
	Some college^b^	N/A
**Monthly income**		
	Low (under median)	0.59
	High (median or above)	1.18
	None or don’t know^b^	N/A
**ART^d^ adherence**		
	High	1.51
	Not using, low, or neutral^b^	N/A
**Substance use^e^**		
	Alcohol	1.29
	Tobacco	1.62
	Marijuana	1.70

^a^OR: odds ratio.

^b^Reference category.

^c^N/A: not applicable.

^d^ART: antiretroviral therapy.

^e^Reference category was none for each substance.

Self-reported high ART adherence was associated with higher odds of seeking GH and SRH information versus low adherence or not being on ART (OR 1.62 and 1.51, respectively). White ethnicity was associated with higher odds of seeking SRH information versus other racial/ethnic groups (OR 1.68). Male gender identity was associated with higher odds of seeking GH and SRH information (OR 1.19 and 1.26, respectively) and transgender gender identity was associated with lower odds of seeking GH and SRH information (OR 0.41 and 0.50, respectively) relative to other gender identities. The odds of seeking SRH information online were approximately twice as high for those reporting “other” as their sexual orientation (ie, excluding those identifying as gay, lesbian, bisexual, or straight) (OR 2.48).

## Discussion

### Principal Findings

This study is among the first to report internet health information–seeking behaviors among YALH. We found that a significant minority of YALH used the internet to find GH (211/716, 29.5%) and SRH information (155/716, 21.6%). The rates of technology use and health information seeking were similar in this population to previous reports of predominantly racial/ethnic minority samples of homeless youth, who may face many similar challenges [30].

Patterns of seeking health information were associated with several demographic factors. As reported in the general population [8], YALH in this sample with higher socioeconomic status (ie, education, income) were more likely to go online to seek information regarding both GH and SRH. Interestingly, reporting a sexual orientation of “other” as opposed to gay, straight, or bisexual was also associated with increased SRH information seeking. This may reflect that adolescents and young adults who are exploring their sexuality may feel more comfortable finding health information online [31] as opposed to seeking health information from a person (eg, provider) due to perceived or enacted stigma in the health care setting [32].

Also consistent with general population findings [2], health-related information seeking in the sample was most likely to be directed toward GH topics, like diet and exercise. This focus likely stems from progress in HIV treatment and care [33] and highlights the importance of providers focusing on holistic health. Fewer YALH searched for SRH, most commonly to explore STI symptoms, testing, and treatment. Among transgender individuals (mostly transgender women), nearly a quarter searched for information about hormones, surgery, or other procedures. This is particularly important given poor access to gender-affirming services experienced by this population [34] and underscores the need for integration of gender-affirming care with HIV prevention and treatment services.

### Limitations

Several study limitations should be noted. We attempted to engage young HIV-positive individuals who were struggling with adherence and engagement in care. However, this sample did not include those who are disengaged or lost to care, possibly due to syndemic health issues. This group may have very different internet health information–seeking patterns. It is also important to acknowledge that our sample was recruited to participate in digital HIV interventions, suggesting a higher proportion of YALH who seek health information on the internet than the general population of YALH. Further, while youth were recruited from 10 sites across the United States, regional differences in service options and HIV-related stigma may differentially affect YALH (ie, youth living in more rural areas). Region was not retained as a predictor in the final model, but regional differences may not have been adequately captured by study site locations or the regional predictor variable that we created.

### Conclusions

Despite these limitations, this is one of the first studies to address internet health information–seeking behaviors among a marginalized group of youth living with a chronic disease. High rates of internet use among YALH and nearly one-quarter of participants seeking health information online have important implications for clinicians and health educators working with YALH and other marginalized populations. Health care providers should receive training in how to engage in open discussions with patients about their technology use, the SRH topics they search for, and ways of ensuring the information being accessed online is reputable. These direct discussions may help reduce stigma and be particularly useful in supporting transitional age youth. Transitioning from pediatric to adult HIV care is commonly associated with poor retention in care [35]; leveraging eHealth literacy support represents an opportunity to improve care outcomes during this period.

While measures related to eHealth literacy (ie, YEHS and MTUAS subscales) were not retained in models, interventions to build transactional eHealth literacy skills (ie, skills to locate and understand, exchange, evaluate, and apply health information [36]) among YALH may still strengthen their engagement in care and increase access to high-quality health information via trusted communication channels (eg, governmental organizations), even through social networking platforms [37,38]. Though user-generated health information content shared on social networking platforms may not be as accurate or trustworthy as scientific or governmental sources, there is value in social networking platforms and tools regarding reach and engagement. Widening disparities in the quality of health information online, particularly on popular social media, may compromise the adoption of these platforms by trusted creators of online health information. For example, though the CDC maintains accounts on legacy social media networks (eg, Instagram), newer technologies can quickly lure younger adults away from carefully crafted messaging. To meet this growing need for trustworthy health information online that serves YALH, creators of digital health information need to be innovative in developing strategies for meeting YALH where they are online.

## Abbreviations

ACASI: audio computer-assisted self-interview
ART: antiretroviral therapy
AUC: areas under the receiver operating curve
GH: general health
HHS: US Department of health and Human Services.
ML: machine learning
MTUAS: Media and Technology Usage and Attitude Scale
OR: odds ratio
ROC: receiver operating curve
SRH: sexual and reproductive health
UCLA: University of California, Los Angeles
YALH: young adults living with HIV
YEHS: youth engagement with health services
